# Effect of Cu Intercalation Layer on the Enhancement of Spin-to-Charge Conversion in Py/Cu/Bi_2_Se_3_

**DOI:** 10.3390/nano12203687

**Published:** 2022-10-20

**Authors:** Shu Hsuan Su, Cheong-Wei Chong, Jung-Chuan Lee, Yi-Chun Chen, Vyacheslav Viktorovich Marchenkov, Jung-Chun Andrew Huang

**Affiliations:** 1 Department of Physics, National Cheng Kung University, Tainan 701401, Taiwan; 2 Sheng Chuang Technology Company, Taichung 407330, Taiwan; 3 M.N. Miheev Institute of Metal Physics, UB RAS, 620108 Ekaterinburg, Russia; 4 Institute of Physics and Technology, Ural Federal University, 620002 Ekaterinburg, Russia; 5 Department of Applied Physics, National University of Kaohsiung, Kaohsiung 811726, Taiwan; 6 Taiwan Consortium of Emergent Crystalline Materials, National Science and Technology Council, Taipei 10622, Taiwan

**Keywords:** spin-to-charge conversion, inverse Edelstein effect, topological insulator, spin pumping

## Abstract

The spin-to-charge conversion in Permalloy (Py)/Cu/Bi_2_Se_3_ is tunable by changing the Cu layer thickness. The conversion rate was studied using the spin pumping technique. The inverse Edelstein effect (IEE) length λ_IEE_ is found to increase up to ~2.7 nm when a 7 nm Cu layer is introduced. Interestingly, the maximized λ_IEE_ is obtained when the effective spin-mixing conductance (and thus J_s_) is decreased due to Cu insertion. The monotonic increase in λ_IEE_ with decreasing J_s_ suggests that the IEE relaxation time (τ) is enhanced due to the additional tunnelling barrier (Cu layer) that limits the interfacial transmission rate. The results demonstrate the importance of interface engineering in the magnetic heterostructure of Py/topological insulators (TIs), the key factor in optimizing spin-to-charge conversion efficiency.

## 1. Introduction

Traditional electronic devices use electrical charge and voltage to process and read information. In addition, spin-based electronic devices use electron spins to carry information. The term “spin-to-charge conversion” essentially refers to the ability of materials to link the electrical charge for information-processing with the use of electron spins. In other words, efficient spin-to-charge conversion permits the effective exchange of spin currents into voltages, allowing electronic devices to easily read information. Spin-to-charge conversion can be achieved in Rashba systems and topological insulators (TIs) through the IEE [1,2]. Among these materials, a TI-based heterostructure is a prospective candidate for ultrahigh inverse Edelstein effect length (*λ*_IEE_) due to its unique surface states with spin-momentum locking [3,4]. Moreover, highly efficient spin-to-charge conversion can be used in logic devices such as the magneto-electric spin-orbit (MESO) device [5,6]. Therefore, the enhancement of spin-to-charge conversion in TIs is suitable for readout schemes in many advanced spintronic devices.

Due to the spin momentum locking, the 3D spin current density J_s_ injected onto the TI surface will produce two-dimensional (2D) charge density $J_{c}^{2D}$ on the TI surface states (SS), the so-called IEE. The IEE length (λ_IEE_) is determined to be $J_{c}^{2D}$/J_s_, which can be experimentally probed using the spin pumping technique [7,8,9,10,11,12]. Numerous studies have been carried out to determine the spin-to-charge conversion efficiencies in 3D Tis [3,4,7,10,12]. In particular, a spin Hall angle (SHA) as large as ~0.43 has been reported in Bi_2_Se_3_, which is attributed to the enhanced spin current through surface states which is then converted into DC-voltage due to bulk inverse spin Hall effect (ISHE) [11]. However, large variations in the SHA were found, in an order of magnitude difference, which the authors attribute to the inhomogeneity of the interface quality [11].

On the other hand, a dominant role for surface states in spin-to-charge conversion was observed, despite the unavoidable conducting bulk in Bi_2_Se_3_ [7]. Although the thickness of Bi_2_Se_3_ varied from 2 QL to 60 QL, the effective spin-mixing conductance does not increase monotonically, suggesting that surface states dominated the mechanism [7]. Clearly, the spin pumping characteristics are an important parameter to study the spin-to-charge conversion mechanism in 3D TIs, where controlling the interfacial properties is a necessary step [4,11].

To improve the spin-injection efficiency via the ISHE, it has been proposed to use an intercalator (e.g., Cu or Ag) as a potential barrier between the ferromagnets (FM) and the heavy metal (HM) [13,14]. This is effective in tuning the interfacial spin-dependent resistivity and improving the spin injection. Cu is widely used to control the spin transmissivity in multilayer devices [13,15,16]. Du et al., demonstrated that the insertion of a Cu layer between Y_3_Fe_5_O_12_ (YIG) and W substantially improved the spin current injection into W, while a similar insertion between YIG and Pt degraded the spin current [13]. The authors reported a quantitative analysis finding that the spin transport efficiency in heterostructures depends on the spin conductance of each component and their interfaces [13]. Similar results were reported by Deorani et al., where the effect of the Cu interlayer on spin-mixing conductance does depend on the materials (Pt versus Ta) [16]. The spatial mapping of spin accumulation in Cu due to the spin-pumping effect was observed using scanning transmission X-ray microscopy [17]. Recently, Cu layers have been deposited on TI films to eliminate proximity-induced ferromagnetism in spin-orbit torque (SOT) devices [18]. In particular, the deposition of Cu on TI may play a minor role in changing the surface-state conditions, such as causing an energy shift of the Dirac point and valence-band maximum, since the work function of Cu and the electron negativity of TI are of similar magnitudes [19]. Although Cu is the most commonly used spacer layer in the spintronic devices, there is still a lack of quantitative studies on the role of Cu insertion in the spin-to-charge conversion in TIs that measured based on a spin-pumping mechanism.

**Figure 1:**
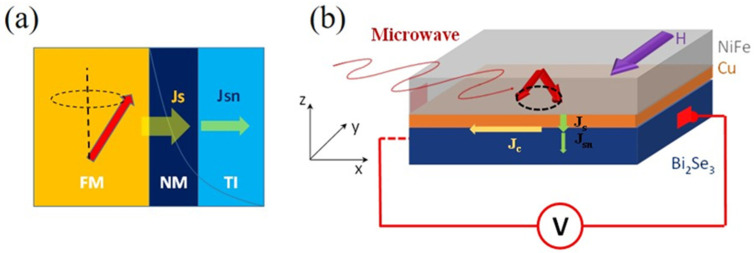
Schematic of spin pumping measurements of tri-layer samples, with a nonmagnetic (NM) spacer introduced in between the FM and TI layer. Upon microwave excitation, the magnetization of the FM layer processes and pumps a pure spin current into the NM layer and induces a charge current via the IEE. Due to the potential barrier at the NM interface, the pumped spin current J_S_ is partially depleted at the interface, and only part of the spin current J_SN_ propagates in the TI layer. (**a**) Cross-sectional view. (**b**) 3D view.

In this work, we fabricated a trilayer structure of Py/Cu/Bi_2_Se_3_ and studied the spin-pumping characteristic by varying the thickness of the Cu layer (as shown in Figure 1a,b). The Cu layer was used to protect the TI surface from exchange interaction with Py. Our results show that Cu also acts as a barrier for spin transport into the TI film. More importantly, the spin-to-charge conversion efficiency was enhanced due to the introduction of the Cu barrier. The related mechanism is discussed in this work.

## 2. Experimental

Bi_2_Se_3_ films with a thickness of 10 nm were synthesized using the molecular beam epitaxy (MBE) method [10]. The structural characterization of Bi_2_Se_3_ thin films is investigated by X-ray diffraction (XRD) and atomic force microscopy (AFM) (see Section 2 of the Supplementary Information). The as-grown Bi_2_Se_3_ were in situ capped with a 2 nm Se layer, which was used as a protective layer. The samples were then transferred into a pulsed laser deposition (PLD) chamber for deposition of Cu and subsequently Ni_80_Fe_20_ (Py) layers at room temperature. Before depositions, the Se layer was decapped in the PLD chamber at about 180 °C for 1 h. A series of trilayer samples were prepared by varying the thickness of Cu from 3 to 11 nm. The quality of the Py/Cu/ Bi_2_Se_3_ trilayer was examined by high-resolution transmission electron microscopy (HRTEM), as shown in Figure S3c in the Supplementary Information). AvPy/Bi_2_Se_3_ bilayer was also prepared for comparison. The Py thickness was fixed at 17 nm. A 1 nm of Al film was deposited on Py as a capping layer. To evaluate the spin-to-charge conversion, a spin-pumping technique was utilized (Figure 1). A spin current was generated in Py via its ferromagnetic resonance (FMR) condition and injected into Bi_2_Se_3_, passing through the Cu-inserted layer (-z direction) (Figure 1b). The DC voltage was measured in the x-direction and the resulting 2D charge current can be evaluated [12]. All measurements were performed at room temperature.

## 3. Results and Discussion

**Figure 2:**
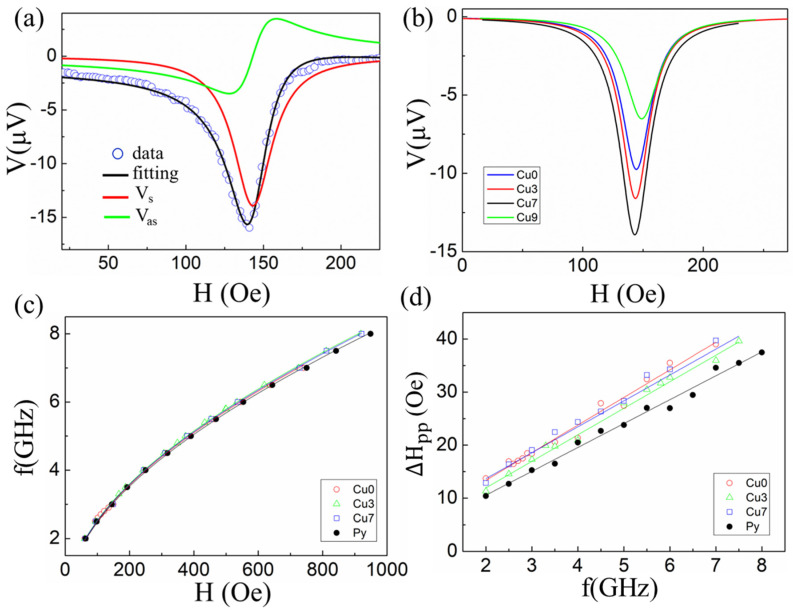
(**a**) DC voltage measured at 3GHz for Py/Cu (7nm)/Bi_2_Se_3_; (**b**) V_s_ extracted for various samples (Cu0, Cu3 and Cu7 denote t_Cu_ = 0, 3, 7 nm respectively); (**c**) excitation frequency as a function of the resonant field. The solid lines are the curves fitted using Kittel formula; (**d**) frequency dependence of FMR linewidths for samples with different Cu thicknesses. The solid lines show the linear fit from which the damping factor (α) of each sample is derived.

Figure 2a shows the spin-pumping voltage as a function of the magnetic field (H) measured at an excitation frequency of 3 GHz for the sample Py/Cu (7nm)/Bi_2_Se_3_. The results of other frequencies and magnetic field sweeps can be found in Figures S1 and S2 in the Supplementary Information. The voltage signals consisting of symmetric (V_s_) and antisymmetric (V_as_) parts can be isolated by fitting the measured voltage (data curve) to the form

(1) $$V=\frac{V_{s}{\left(\Delta H\right)}^{2}}{\left({\left(\Delta H\right)}^{2}+{\left(H-H_{r}\right)}^{2}\right)}+\frac{V_{as}\left(\Delta H\left(H-H_{r}\right)\right)}{\left({\left(\Delta H\right)}^{2}+{\left(H-H_{r}\right)}^{2}\right)}$$

Here $H_{r}$ is the FMR resonant field and ΔH is the line width of the signal. The obtained DC voltage signals consist of several components of the IEE, the inverse spin Hall effect (ISHE) [13,14,20], the shunting effect [13,21], the spin rectification effects (including the anomalous Hall effect (AHE) and the anisotropic magnetoresistance (AMR)) [21,22]. The contributions of the IEE and ISHE are related to the $V_{s}$ of the resonance field. However, due to the shunting effect of the Py layer and the overlapping symmetrical AMR and AHE signals in the excitation geometry, the relative weights of the contribution of the voltage generated by the IEE to the total Vs cannot be quantitatively separated [23,24]. Moreover, the voltage polarization of our results is similar to other Bi_2_Se_3_-based spin pumping [7,25,26]. Similar fitting was also done here, and $V_{s}$ was extracted as shown in Figure 2b. It was found that Vs is larger in the presence of a Cu layer. FMR experiments were also conducted as shown in Figure 2c,d.

The FMR linewidth ($\Delta H_{pp}$) of samples with different Cu thicknesses was plotted as a function of the excitation frequency for the Py/Cu/Bi_2_Se_3_ samples in Figure 2d. The damping factor (α) was obtained by fitting $\Delta H_{pp}$ to f using this formula, $\Delta H_{pp}=H_{0}+\frac{4\pi }{\gamma }\alpha f$, in which $\;H_{0}$ corresponds to the presence in the Py layer [13,14]. Compared with single Py, the linear fitted slopes are larger for the Py/Bi_2_Se_3_ bilayer and Py/Cu/Bi_2_Se_3_ trilayer samples, indicating that the injection of the spin current into Bi_2_Se_3_ results in the broadening of the FMR linewidth and thus larger damping constants α. Interestingly, α_Py/Cu/Bi2Se3_ was found to decrease from $\left(1.262\pm 0.05\right)\times 10^{-2}$ to $\left(1.185\pm 0.05\right)\times 10^{-2}$ when the thickness of the Cu layer was increased to 7 nm.

**Figure 3:**
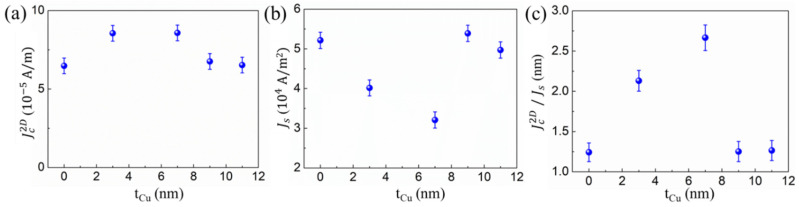
(**a**) $J_{c}^{2D}$ vs. t_Cu_; (**b**) J_s_ vs. t_Cu_; (**c**)$\;J_{c}^{2D}$ /J_s_ vs. t_Cu_ measured at 3GHz excitation frequency.

The resistance of the multilayer samples R_d_ was measured using a four-probe method. $J_{c}^{2D}$ was determined as $J_{c}^{2D}$= I_c_/w = V_s_/wR_d_, where w and I_c_ are the width of the sample and charge current, as shown in Figure 3a. The estimated $J_{c}^{2D}$ was derived from the 2D charge current in the x-direction at the interface via the inverse Edelstein effect (IEE), the charge current induced by the ISHE of the Cu layer [27], and the current derived from the ferromagnetic transport in the Py layer. We evaluated the spin-to-charge conversion $J_{c}^{2D}$/J_s_. using standard analysis of spin pumping on TI [7,10,12]. The spin-mixing conductance $G_{eff}^{↑↓}$ used to account for the efficiency of generating the spin current was extracted using Equation (2):

(2) $$G_{eff}^{↑↓}=\frac{4\pi M_{s}t_{Py}}{g\mu_{B}}\Delta \alpha$$

where M_s_ is the saturation magnetization of Py, t_Py_ is the thickness of Py, g is the Landé factor and u_B_ is the Bohr magneton. M_s_ was calculated from *f* vs. H_r_ using the Kittel formula, $f=\frac{\gamma }{2\pi }\sqrt{H_{r}\left(H_{r}+4\pi M_{eff}\right)}$, in which γ is the gyromagnetic ratio to extract the effective saturation magnetization ($M_{eff}$) (Figure 2c) [10,12,13]. Δα = α_Py/Cu/Bi2Se3_ − α_Py_ and is determined by analyzing ΔH_pp_ vs. *f*, as shown in Figure 2d. For the spin current densities injected through the interface due to spin pumping, Equation (3) was utilized as follows:

(3) $$J_{s}^{3D}=\frac{G_{eff}^{↑↓}{}^{2}\gamma^{2}\hbar h_{rf}{}^{2}}{8\pi \alpha^{2}}\left[\frac{4\pi M_{s}\gamma +\sqrt{{\left(4\pi M_{s}\gamma \right)}^{2}+4\omega^{2}}}{{\left(4\pi M_{s}\gamma \right)}^{2}+4\omega^{2}}\right]\left(\frac{2e}{\hbar }\right)$$

in which *γ* is the gyromagnetic ratio, ω(=2πf) is the frequency, and h_rf_ is the amplitude of the microwave *rf* field $.{\;h}_{\mathrm{rf}}=\frac{I_{\mathrm{rf}}}{2w}$, [10,11,12] in which $I_{rf}$ is the microwave current at a frequency of 3 GHz and w is the linewidth of the coplanar waveguide, respectively. $h_{\mathrm{rf}}$ is estimated to be 0.112 Oe for $I_{rf}=0.0178\;A\;\mathrm{and}\;w=1\;\mathrm{mm}$. The calculated J_s_ is presented in Figure 3b. By dividing $J_{c}^{2D}$ with J_s_, the spin-to-charge conversion efficiency $J_{c}^{2D}$/J_s_ (λ_IEE_) can be determined.

**Figure 4:**
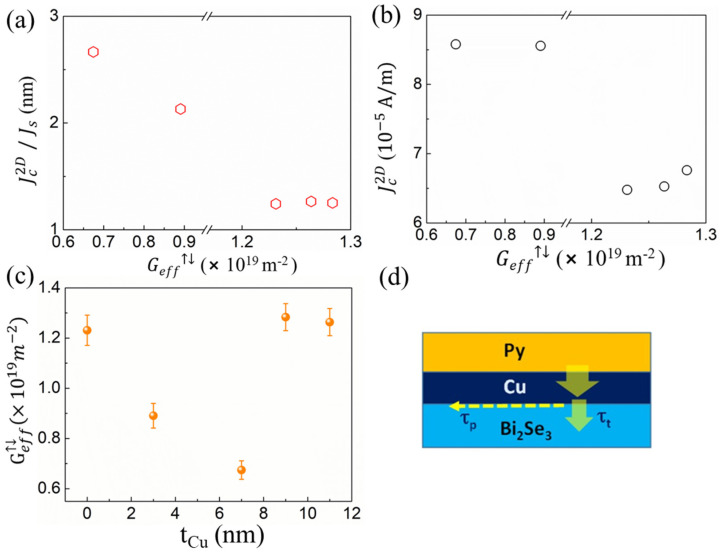
(**a**) $J_{c}^{2D}$/J_s_ at various $G_{eff}^{}$ (Py/Cu/TI); (**b**) $J_{c}^{2D}$ at various $G_{eff}^{}$ (Py/Cu/TI); (**c**) $G_{eff}^{}$ (Py/Cu/TI) vs. t_Cu_; (**d**) schematic illustrates the spin transport in Py/Cu/TI.

Figure 3a plots the $J_{c}^{2D}$ versus t_Cu_. There is an optimized $J_{c}^{2D}$ at a thickness of 3 nm and 7 nm. In contrast, J_s_ decreases when 3 and 7 nm Cu are added, as shown in Figure 3b. The variation trend of J_s_ vs t_Cu_ is consistent with the change in the effective spin-mixing conductance $G_{\mathrm{eff}}^{↑↓}$, where the $G_{\mathrm{eff}}^{↑↓}$ is found to decrease with the introduction of 3 and 7 nm Cu in Figure 4c. The variation in $G_{\mathrm{eff}}^{↑↓}$ is discussed below. Interestingly, a maximized $J_{c}^{2D}$/J_s_ is observed at t_Cu_ = 7 nm, where λ_IEE_ reaches ~2.7 nm, as shown in Figure 3c. This result suggests that the optimization of $J_{c}^{2D}$/J_s_ may be related to the reduction of J_s_ due to the Cu insertion. In addition, the value of λ_IEE_ = 1.25 nm for the Py/Bi_2_Se_3_ without the Cu insertion sample, which is larger than that previously reported on FM/Bi_2_Se_3_ systems [7,10,11].

To investigate the possible reason for the enhancement of $J_{c}^{2D}$/J_s_, we plotted $J_{c}^{2D}$/J_s_ as a function of the effective spin-mixing conductance $G_{eff}^{↑↓}$ (Py/Cu/TI) as shown in Figure 4a. Various $G_{eff}^{↑↓}$ (Py/Cu/TI) values were obtained by changing the Cu layer thickness. Large $r\;J_{c}^{2D}$/J_s_ are obtained at low values of $G_{eff}^{↑↓}$ (Py/Cu/TI) (hence the minimum J_s_ as shown in Figure 3b). We further examined $J_{c}^{2D}$ vs. $G_{eff}^{↑↓}$ (Py/Cu/TI), as shown in Figure 4b. $J_{c}^{2D}$ does not increase with the increasing $G_{eff}^{↑↓}$ (Py/Cu/TI), revealing that the spin-to-charge mechanism may not be dominated by the bulk spin Hall effect (SHE) [16]. Therefore, we propose here that the spin-to-charge conversion in the Py/Cu/Bi_2_Se_3_ system arises from the IEE, where the origin is the spin-momentum locked surface states of the TI layer, as explained in other literatures [7,25,26].

Low $G_{eff}^{↑↓}$ (Py/Cu/TI) indicates a strong spin backflow and spin memory loss (spin absorption) at the high SOC interface [28,29]. Both factors are relevant in this Py/Cu/TI trilayer system. If we examine $G_{eff}^{↑↓}$ (Py/Cu/TI) at various t_Cu_, as presented in Figure 4c, except for Py/Cu (3 nm)/TI and Py/Cu (7 nm)/TI, the samples Py/TI, Py/Cu (9 nm)/TI and Py/Cu(11 nm)/TI exhibit $G_{eff}^{↑↓}$ (Py/Cu/TI) ~1.25 × 10^19^ m^−2^, which is typical for metal–metal interfaces [30,31]. As reported by Du et al., the effective spin-mixing conductance of the trilayer system (FM/Cu/NM, FM for ferromagnetic, while it is NM for nonmagnetic material) is determined by the serial contribution of the two interfaces (FM/Cu and Cu/NM) and the spin resistance of Cu [13]. Here we refer to FM as Py and NM as the TI film; the $G_{eff}^{↑↓}$ (Py/Cu/TI) can be as described by Equation (4):

(4) $$\frac{1}{G_{eff}^{↑↓}\left(\mathrm{Py}/\mathrm{Cu}/\mathrm{TI}\right)\;}=\frac{1}{G_{Py/Cu}^{↑↓}}+R_{Cu}+\frac{1}{G_{Cu/TI}}$$

where $G_{Py/Cu}^{↑↓}$ is the spin-mixing conductance of the Py/Cu interface, $R_{Cu}\;$ is the spin resistance and $G_{Cu/TI}$ is the spin conductance of Cu/TI. One of the reasons for the lower $G_{eff}^{↑↓}$ (Py/Cu/TI) compared to $G_{Py/Cu}^{↑↓}$ may be due to the fact that the $G_{Cu/TI}$ is smaller than that of $G_{Py/TI}^{↑↓}$, similar to the case in Cu/Pt [13,16]. However, since $G_{eff}^{↑↓}\left(\mathrm{Py}/\mathrm{Cu}/\mathrm{TI}\right)$ ≈ $G_{Py/Cu}^{↑↓}$ at t_Cu_ ≥ 9 nm, here we assume that Cu/TI and Py/Cu exhibit similar qualities to $G_{Py/Cu\;}^{↑↓}$ ≈ $G_{Cu/TI}$. Thus, by assuming that the degree of spin absorption at the Cu/TI interfaces is similar in all cases, we suggest that the reason for the lower $G_{eff}^{↑↓}\left(\mathrm{Py}/\mathrm{Cu}/\mathrm{TI}\right)$ of 3 nm and 7 nm Cu-based trilayer samples may be due to the strong spin accumulation at this ultrathin regime [13]. When the Cu layer is too thin, Py/Cu does not cause significant damping enhancement due to the poor spin sinking of Cu. The spin accumulation in Cu leads to a backflow into Py; therefore, $G_{eff}^{↑↓}\left(\mathrm{Py}/\mathrm{Cu}/\mathrm{TI}\right)\;$ is much smaller than $G_{Py/Cu}^{↑↓}$. The spin accumulation is uniform throughout the Cu buffer layer. The spin pumping will now be partitioned. Some of the pumped spins are reflected to the FM, while the rest are transmitted and relaxed in the TI layer. The spin-accumulation-driven current is significant for light metals or metals with only *s* electrons in the conduction band, and their spin-flip to spin-conserving scattering ratios are very small [15]. This feature may strongly affect the final performance of the spin-pumping efficiency. Therefore, compared to t_Cu_ ≥ 9 nm, a stronger spin backflow occurs, which eventually leads to a decrease in $G_{eff}^{↑↓}\left(\mathrm{Py}/\mathrm{Cu}/\mathrm{TI}\right)$. According to recent reports [32,33], $G_{eff}^{↑↓}\left(\mathrm{Py}/\mathrm{Cu}/\mathrm{TI}\right)\;$ changes with the increase in Cu thickness, which may be attributed to the oscillatory behavior caused by the quantum well state in the NM layer [32] and the magnetic anisotropy induced by the interlayer coupling in Py/Cu [33].

The decrease in $G_{eff}^{↑↓}\left(\mathrm{Py}/\mathrm{Cu}/\mathrm{TI}\right)\;$ seems have strong correlation with the spin-to-charge conversion efficiency. The next question is how such a condition could increase $J_{c}^{2D}$/J_s_? Here we defined $J_{c}^{2D}$/J_s_ as λ_IEE_ = ν*_F_* τ where ν*_F_* is the Fermi velocity of the TI surface states and τ is the IEE relaxation time. As shown in the Figure 4d, τ is modified due to the tunnelling current into the TI, which is determined by the momentum relaxation time τ_p_ and the interface tunnelling time τ_t_ as shown in Equation (5) [34]:

(5) $$\lambda_{IEE}=\frac{\lambda_{mf}}{\left(1+\frac{2\tau_{p}}{\tau_{t}}\right)}$$

where $\lambda_{mf}$ = ν_F_ τ_p_ is the mean free path in the TI. From this model, we propose that the monotonic increase in $\lambda_{IEE}$ and the decrease in $G_{\mathrm{eff}}^{↑↓}$ are attributed to the modification in the IEE relaxation time τ due to the additional tunnelling barrier (Cu) that limits the interfacial transmission rate (1/τ_t_) [34,35]. $\lambda_{IEE}$ is always lower than $\lambda_{mf}$ due to the correction factor of (1+2τ_p_/τ_t_). It is obvious that $\lambda_{IEE}\;$ can be increased by reducing 1/τ_t_, which can be done by introducing a tunnelling barrier in between the Py and TI layers. Using λ_IEE_ (t_Cu_ =7 nm) = 2.7 nm and based on our previous ARPES results, ν*_F_* = 5.7 × 10^5^ m/s [36], we find τ~4.7 *f*s, the same order of magnitude as Bi/Ag [37] and α-Sn/Ag [8] interfaces. Our extracted λ_IEE_ (t_Cu_ = 2.7 nm) is higher than 0.1–0.4 nm in the Bi/Ag Rashba interface [37], 2.1 nm and 2 nm in TI SS of α-Sn/Ag [8] and HgTe/HgCdTe [9], respectively. We attribute the enhancement to the insertion of the Cu tunnelling barrier. Although more theoretical calculations may be needed, our work demonstrates the importance of interface engineering to enhance the spin-to-charge conversion.

This method can also be applied to other high-SOC interfaces to obtain a high spin-to-charge conversion based on the inverse Edelstein effect, which is critical for spin current detectors and other novel applications such as broadband terahertz emitters [38,39].

## 4. Conclusions

In conclusion, we investigated the spin-to-charge conversion in Py/Cu/Bi_2_Se_3_ using spin-pumping techniques. Enhancement of $J_{c}^{2D}$/J_s_ with increasing t_Cu_ was observed at room temperature, where $J_{c}^{2D}$/J_s_ ~2.7 nm when a 7 nm of Cu layer was inserted. We proposed that the enhancement is attributed to the additional Cu interlayer acting as a tunnelling barrier that modifies the relaxation time at the interface. This work has provided a feasible route to improving the spin-to-charge conversion efficiency of TIs, which is crucial for the applications of spin functional devices.

## Data Availability

All data included in this study are available upon request by contacting the corresponding author.

## Supplementary Materials

The following supporting information can be downloaded at: https://www.mdpi.com/article/10.3390/nano12203687/s1, Figure S1: Spin-pumping induced voltage in Py/Cu (7 nm)/Bi2Se3 measured at various excitation frequencies; Figure S2: Magnetic field scans of the spin-pumping voltage measured in Py/Bi2Se3 at three different in-plane angles; Figure S3: The structure of Bi_2_Se_3_ film. References [36,40,41] are cited in the Supplementary Materials.
