# Supplementary material for: Effect of Cu Intercalation Layer on the Enhancement of Spin-to-Charge Conversion in Py/Cu/Bi2Se3
Source: Nanomaterials (Basel). 2022 Oct 20;12(20):3687. doi: 10.3390/nano12203687 (PMC9606994; doi:10.3390/nano12203687)
Supplement: Supplementary file 1 [file nanomaterials-12-03687-s001.zip › nanomaterials-1948896-supplementary.pdf]

nanomaterials

Supplementary Material

Effect of Cu Intercalation Layer on the Enhancement of Spin-to-Charge Conversion in Py/Cu/Bi_2Se_3

Shu Hsuan Su [1,*], Cheong-Wei Chong [1], Jung-Chuan Lee [1,6], Yi-Chun Chen [1], Vyacheslav Viktorovich Marchenkov [2,3] and Jung-Chun Andrew Huang [1,4,5,*]

1 Department of Physics, National Cheng Kung University, Tainan 701401, Taiwan
2 M.N. Miheev Institute of Metal Physics, UB RAS, Ekaterinburg 620108, Russia
3 Institute of Physics and Technology Ural Federal University, Ekaterinburg 620002, Russia
4 Department of Applied Physics, National University of Kaohsiung, Kaohsiung 811726, Taiwan
5 Taiwan Consortium of Emergent Crystalline Materials, National Science and Technology Council, Taipei 10622, Taiwan
6 Sheng Chuang Technology Company, Taichung 407330, Taiwan
* Correspondence: macg0510@yahoo.com.tw (S.H.S.); jcahuang@mail.ncku.edu.tw (J.-C.A.H.)

Citation: Su, S.H.; Chong, C.-W.; Lee, J.-C.; Chen, Y.-C.; Marchenkov, V.V.; Huang, J.-C.A. Effect of Cu Intercalation Layer on the Enhancement of Spin-to-Charge Conversion in Py/Cu/Bi2Se3. *Nanomaterials* **2022**, *12*, 3687. https://doi.org/10.3390/xxxxx

Academic Editor: Ze Don Kvon

Received: 17 September 2022
Accepted: 17 October 2022
Published: 20 October 2022

Publisher's Note: MDPI stays neutral with regard to jurisdictional claims in published maps and institutional affiliations.

Copyright: © 2022 by the authors. Licensee MDPI, Basel, Switzerland. This article is an open access article distributed under the terms and conditions of the Creative Commons Attribution (CC BY) license (https://creativecommons.org/licenses/by/4.0/).

Section 1. The spin pumping voltage for various excitation frequencies and magnetic field scans

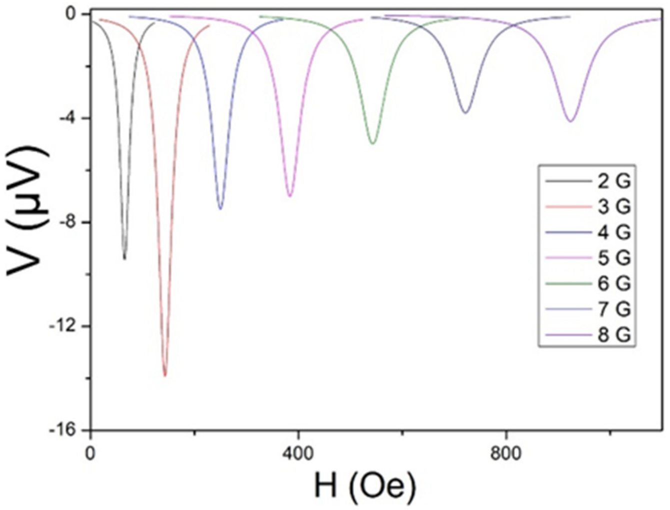

Figure S1. Spin pumping induced voltage in Py/Cu(7nm)/Bi2Se3 measured at various excitation frequencies. The obtained V decreases with the increase of frequency, which is resulted from variation of the performance of the microwave transmission line at different frequencies.

Figure S2. shows spin pumping voltage obtained by sweeping the magnetic-field (H), measured in the Py/ Bi_2Se_3 sample for three in-plane field direction given by Ø= 0°, 90°and 180°, as illustrated in the inset. As expected from the V ∝ $Js \times \sigma$(σ is the direction of spin polarization, $\sigma \| H$), the V values are maximum for Ø= 0° and 180° and V value almost vanishes as Ø= 90°. When magnetic field was reversed (Ø= 0° and 180°), the observed spin pumping signal changed sign as expected from the IEE, indicating that the spin accumulation is reversed on the edge on changing the direction of the magnetic field. It further provides evidence of the IEE induced from spin-momentum locked surface state in Bi_2Se_3.

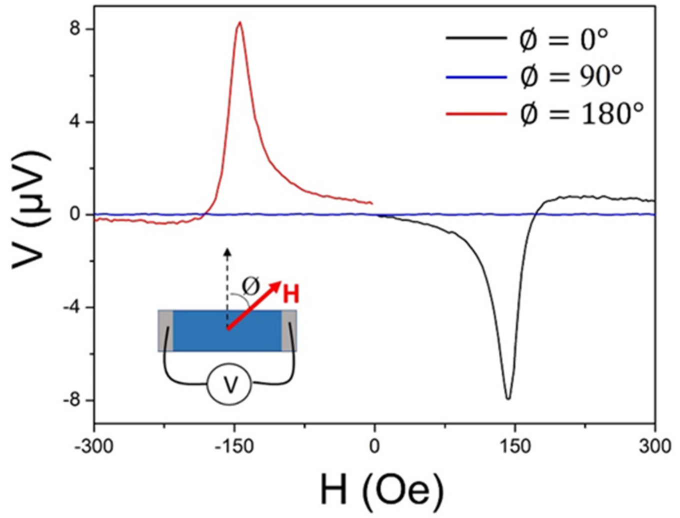

Figure S2. Magnetic field scans of the spin pumping voltage measured in Py/Bi_2Se_3 at three different in-plane angles as illustrated in the inset.

Section 2. The structural characterization of Bi_2Se_3 thin films and Py/Cu/ Bi_2Se_3 trilayer

The structural characterization of Bi_2Se_3 thin films was performed by X-ray diffraction (XRD) and atomic force microscopy (AFM). Fig. S3(a) shows the X-ray θ-2θ scans of the samples. Several features collinear with the direction (00l) were observed, indicating that the single crystal films were grown along the c-axis direction. Large-area surfaces of Bi_2Se_3 films were observed with AFM measurement (in Fig. S3(b)). Large and flat terraces and straight steps are the characteristics of high-quality Bi_2Se_3 film. The triangular shape of the islands is consistent with the lattice symmetry of Bi_2Se_3 (0001) surface. [1, 2] The measured line profile of the height in Fig. S3(d) (corresponding to the red line in Fig. S3(b)) shows that the step height is 1.0 nm, corresponding to the quintuple layer (QL) of Bi_2Se_3 structure. [2, 3] The reflection high-energy electron diffraction (RHEED) shows sharp streaky 1×1 pattern, indicating high quality of the Bi_2Se_3 (0001) surface (inset of Fig. S3(b)), which corresponds well to the AFM data. We also applied high-resolution transmission electron microscopy (HRTEM) to examine the quality of Py/Cu/ Bi_2Se_3 trilayer. Fig. S3(c) shows the HRTEM image along the c axis, clearly showing the epitaxial growth of the Bi_2Se_3 film. It can be seen that the interface is flat and continuous, which is important for effective spin pumping.

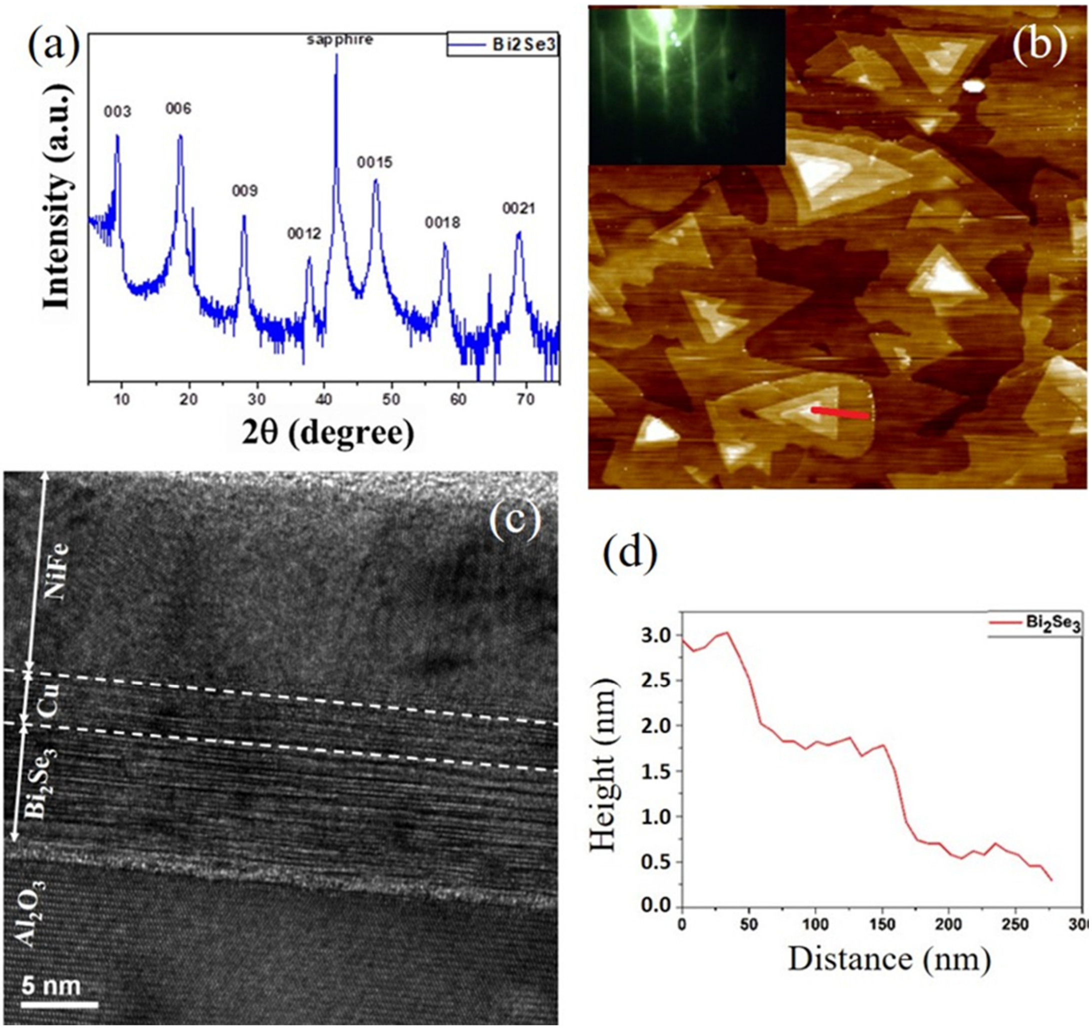

Figure S3. The structure of Bi_2Se_3 film. (a) XRD pattern of Bi_2Se_3 film on *c*-plane sapphire substrate (b) AFM image of 10QL Bi_2Se_3 film (scale: 2 μm x 2 μm). The inset shows the corresponding RHEED pattern. (c) TEM cross-sectional image of a Py/Cu (~3 nm)/Bi_2Se_3 sample. (d) Line scan profile corresponding to the red line in (b).

Reference:

1. Cheng, P.; Song, C.; Zhang, T.; Zhang, Y.; Wang, Y.; Jia, J.-F.; Wang, J.; Wang, Y.; Zhu, B.-F.; Chen, X.; Ma, X.; He, K.; Wang, L.; Dai, X.; Fang, Z.; Xie, X.; Qi, X.-L.; Liu, C.-X.; Zhang, S.-C.; & Xue, Q.-K. Landau Quantization of Topological Surface States in Bi_2Se_3. *Phys. Rev. Lett.* **2010**, 105, 076801.
2. Su, S.H.; Chuang, P.-Y.; Chen, S.W.; Chen, H.Y.; Tung, Y.; Chen, W.-C.; Wang, C.-H.; Yang, Y.-W.; Huang, J.C.A.; Chang, T.-R.; et al. Selective Hydrogen etching leads to 2D Bi(111) bilayers on Bi_2Se_3: Large Rashba splitting in topological insulator heterostructure. *Chem. Mater.* **2017**, 29, 8992–9000.
3. Atuchin, V. V.; Golyashov, V. A.; Kokh, K. A.; Korolkov, I. V.; Kozhukhov, A. S.; Kruchinin,V. N.; Makarenko, S. V.; Pokrovsky, L. D.; Prosvirin, I. P.; Romanyuk, K. N.; Tereshchenko, O. E. Formation of Inert Bi_2Se_3(0001) Cleaved Surface. *Cryst. Growth Des.* **2011**, 11, 5507-5514.
